# Environmental context determines the limiting demographic processes for plant recruitment across a species’ elevational range

**DOI:** 10.1038/s41598-020-67602-5

**Published:** 2020-07-02

**Authors:** Dominik Merges, Jörg Albrecht, Katrin Böhning-Gaese, Matthias Schleuning, Eike Lena Neuschulz

**Affiliations:** 1 Senckenberg Biodiversity and Climate Research Centre Frankfurt, Senckenberganlage 25, 60325 Frankfurt am Main, Germany; 2 Department of Biological Sciences, Goethe Universität Frankfurt, Frankfurt am Main, DE Germany

**Keywords:** Ecology, Community ecology, Forest ecology

## Abstract

Plant recruitment is a multi-stage process determining population dynamics and species distributions. Still, we have limited understanding of how the successive demographic processes depend on the environmental context across species’ distributional ranges. We conducted a large-scale transplant experiment to study recruitment of *Pinus cembra* over six years. We quantified the effects of environmental conditions on four demographic processes and identified the most limiting across and beyond the pines’ elevational range over several years. Realized transition probabilities of the demographic processes varied substantially across the species' distributional range. Seed deposition decreased from the lower to the upper elevational range margin by 90%, but this reduction was offset by increased seed germination and seedling survival. Dispersal limitation at the upper range margin potentially stems from unsuitable seed caching conditions for the animal seed disperser, whereas increased seed germination might result from enemy escape from fungal pathogens and favourable abiotic conditions at the upper range margin. Our multi-year experiment demonstrates that environmental context is decisive for the local relevance of particular demographic processes. We conclude that experimental studies identifying the limiting demographic processes controlling species distributions are key for projecting future range dynamics of plants.

## Introduction

Plant recruitment is a complex process where several plant life stages (e.g., seeds, seedlings, saplings, adults) are connected by demographic processes, such as seed dispersal, predation, germination, and survival^1–3^. The overall recruitment probability of a plant is thus given by the product of successive process-specific transition probabilities^1^. Consequently, a failure in one of the demographic processes could limit the entire recruitment process^1,2,4^. The processes linking plant life stages are affected by the environmental conditions at the site where recruitment takes place, and can thereby be highly dependent on the environmental context^5,6^. Furthermore, many demographic processes depend on biotic interactions, such as the dispersal or predation of seeds by animals, or interactions with fungal pathogens or mutualists^7,8^, which in turn are influenced by the environmental context.

**Figure 1:**
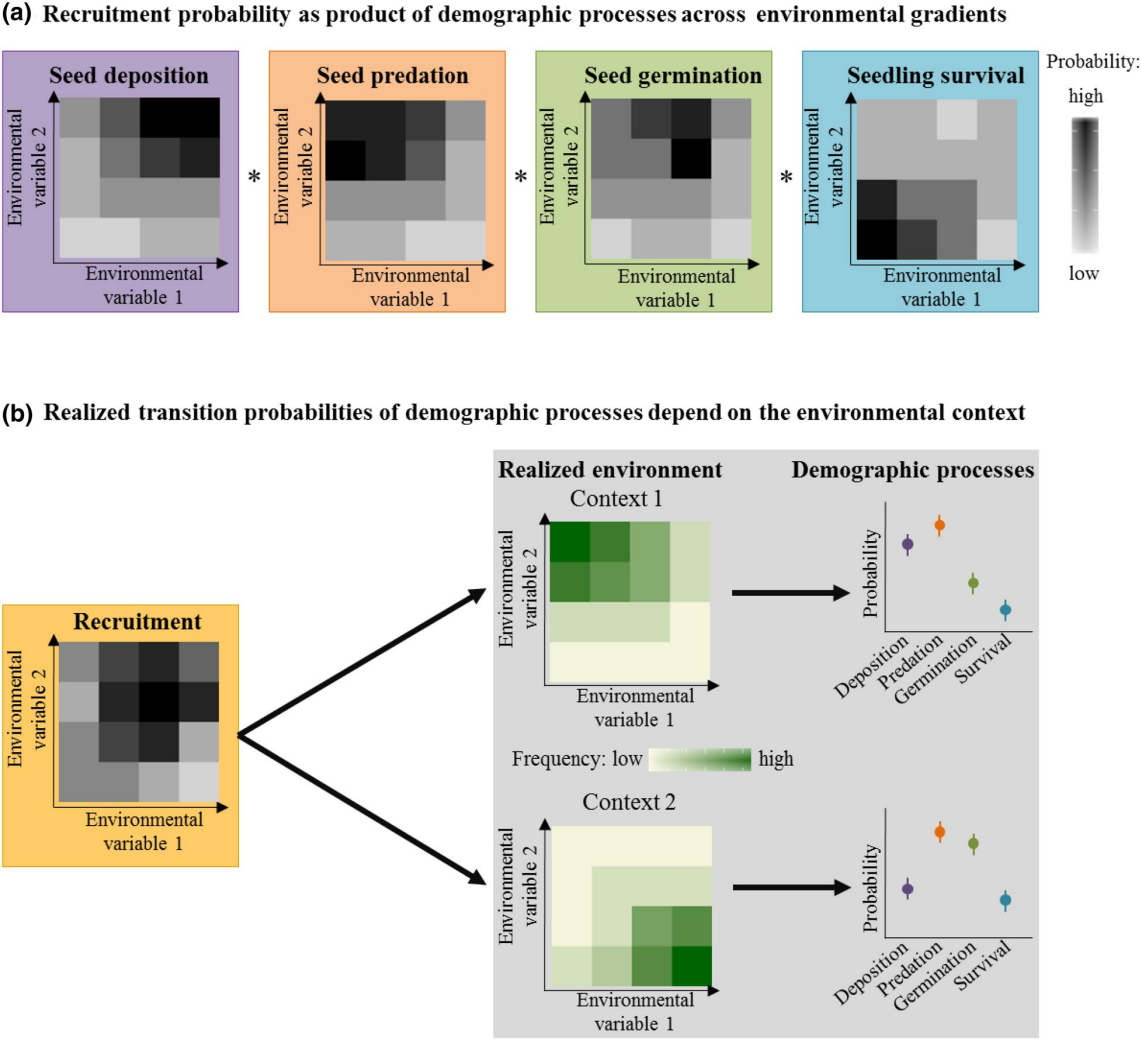
(**a**) The multi-stage process of plant recruitment is the product of seed deposition, the proportion of seeds not predated (i.e., [1-seed predation]), seed germination and seedling survival. Each process varies independently as a function of the environmental context (i.e., environmental variable 1 & 2; grey colour shading). (**b**) Recruitment of a species occurs under different environmental contexts, e.g., environmental conditions may vary between peripheral and central populations across a species range. Consequently, the realized transition probabilities of the individual demographic processes are context-dependent, e.g., compare the hypothetical differences in the frequency of the specific environmental condition between Context 1 & 2 (green colour shading).

Across their distributional ranges, plants are exposed to a substantial variation in the strength of biotic interactions and in abiotic conditions^8–10^, which may alter the demographic processes involved in recruitment (Fig. 1)^11,12^. This context-dependency of plant recruitment is especially important at species’ range margins, where the limitation of a single demographic process may limit a species’ range expansion^12^. Due to the complex interactions between demographic processes, biotic interactions and abiotic conditions, we so far have a very limited understanding of how the environmental context shapes plant recruitment across species distributional ranges^13^. Identifying the limitations and bottlenecks in the demographic processes of plant recruitment across species’ distributional ranges is, however, key for projecting future range dynamics of plants^14,15^.

In this study, we investigate how environmental conditions at different range positions (i.e., at the lower and upper range margin and at the elevational range centre) shape the demographic processes that determine the recruitment of Swiss stone pine (*Pinus cembra*; Fig. 1), a keystone species forming and stabilizing the tree line in parts of the Alps^16^. We assessed four demographic processes (i.e., seed deposition, seed predation, seed germination and seedling survival) of pine recruitment over several years across its elevational range (Fig. 1). The probability of pine recruitment was calculated as the product of the transition probabilities of seed deposition, the proportion of seeds not predated (i.e., 1 − P[seed predation]), seed germination and seedling survival. Seed deposition, seed predation and seed germination were monitored for six years and the survival of seedlings for two years. To assess the context-dependency of each demographic process across the elevational range, we computed the realized transition probabilities of each process by weighting the habitat-specific probabilities with the realized environment at the three range positions. We expected seed deposition to depend on canopy cover and to decrease where canopy cover is absent because the pines’ single seed disperser, the Spotted nutcracker (*Nucifraga caryocatactes* L.), preferentially deposits seeds under closed canopy^17^. Furthermore, we expected high predator densities and seed predation rates under high canopy cover and high ground vegetation cover^5^. Finally, we expected reduced seed germination and survival in dry and shaded conditions^18^ and where specialist fungal pathogens are abundant^19,20^.

**Figure 2:**
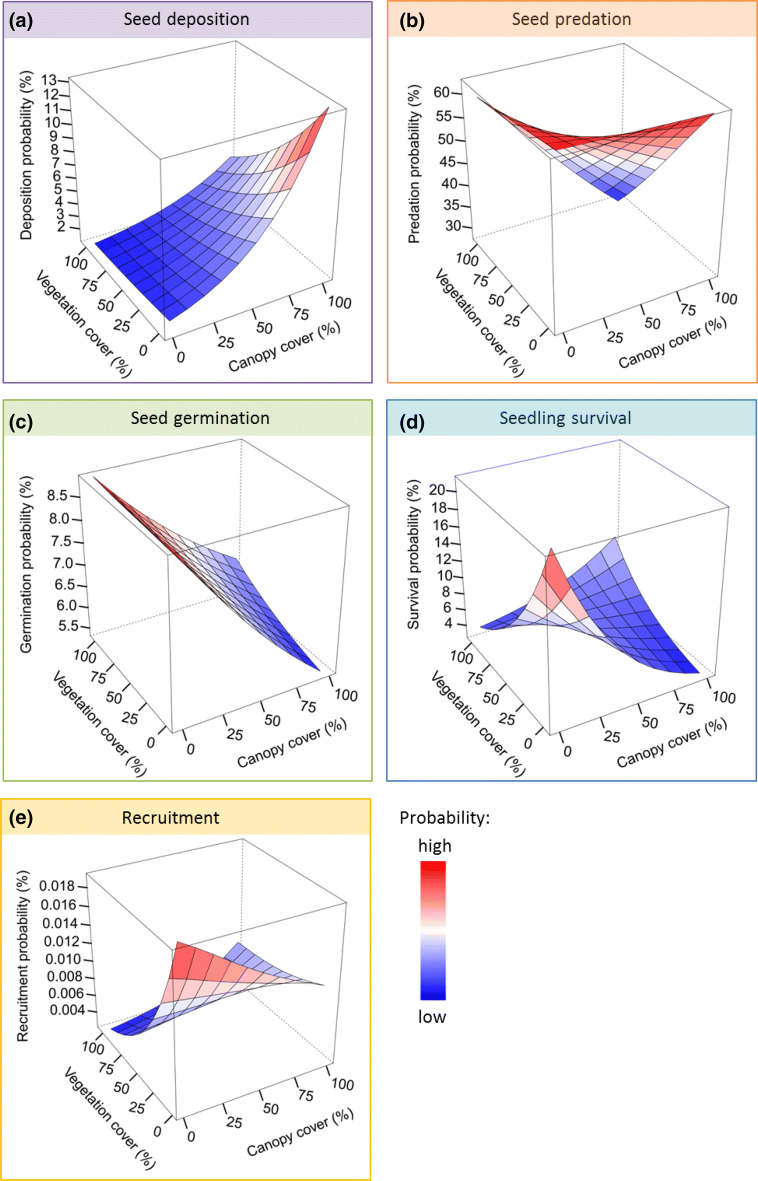
Probability of four demographic processes across the full environmental gradient in terms of canopy cover and ground vegetation cover. (**a**) Probability of seed deposition was highest under closed canopy with no ground vegetation cover (*n*_*obs*_ = 1,381). (**b**) Probability of seed predation was high under all environmental conditions (*n*_*obs*_ = 915). (**c**) Seed germination probability was highest under low canopy cover (*n*_*obs*_ = 2,156). (**d**) Probability of seedling survival was highest under low canopy cover and low ground vegetation cover (*n*_*obs*_ = 319). (**e**) Probability of Swiss stone pine recruitment as product of the predicted probabilities of four demographic processes (i.e., seed deposition, proportion of seeds not predated (i.e., [1-seed predation]), seed germination, seedling survival). Recruitment probability was highest at open microhabitats (i.e., low canopy cover and low ground vegetation cover).

Consistent with our expectations, we found that each demographic process was differently affected along the gradients of canopy and ground vegetation cover (Fig. 2a–d; Table 1). This resulted in the highest overall recruitment probability of 0.02% per sown seed at microhabitats with no canopy or ground vegetation cover (Fig. 2e). In this microhabitat, seed germination and seedling survival were estimated to be highest (Fig. 2c,d; Table 1). Overall recruitment was reduced to 0.01% at sites with high canopy cover and low ground vegetation cover. Under these conditions, low rates of seed germination and seedling survival counteracted the positive effect of high seed deposition rates (Fig. 2a,c,d; Table 1). Overall recruitment was estimated to be lowest (~ 0.002%) at sites without canopy and high ground vegetation cover. At these sites, the combination of low seed deposition and low survival rates, as well as high seed predation rates, overrode the positive effects of high seed germination rates (Fig. 2a–e; Table 1).

**Table 1:** Summary of generalized linear mixed models testing for effects of canopy cover, ground vegetation cover and their interaction on four demographic processes (i.e., seed deposition, seed predation, seed establishment and seedling survival).

Response	Variable	Estimate	2.5% CI	97.5% CI
**Seed deposition** *n*_*obs*_ = 2,156, *n*_*year*_ = 6, *n*_*site*_ = 18	Canopy cover	**0.9**	**0.57**	**1.24**
	Vegetation cover	** − 0.31**	** − 0.55**	** − 0.08**
	Canopy cover × vegetation cover	− 0.14	− 0.39	0.1
	RE year	1.38	0.94	2.14
	RE site	1.19	0.7	2.47
**Seed predation** *n*_*obs*_ = 1,381, *n*_*year*_ = 6, *n*_*site*_ = 18	Canopy cover	0.31	− 0.14	0.77
	Vegetation cover	0.23	− 0.09	0.55
	Canopy cover × vegetation cover	0.22	− 0.06	0.51
	RE year	3.35	3.05	3.69
	RE site	1.94	1.3	3
	RE observation	2.99	1.83	6.05
**Seed germination** *n*_*obs*_ = 915, *n*_*year*_ = 6, *n*_*site*_ = 18	Canopy cover	− **0.21**	− **0.4**	− **0.02**
	Vegetation cover	0.04	− 0.13	0.2
	Canopy cover × vegetation cover	0.01	− 0.14	0.16
	RE year	0.83	0.63	1.04
	RE site	0.23	0	0.48
	RE observation	1.69	1.02	3.45
**Seedling survival** *n*_*obs*_ = 319, *n*_*year*_ = 2, *n*_*site*_ = 18	Canopy cover	− 0.43	− 0.98	0.07
	Vegetation cover	− 0.26	− 0.67	0.13
	Canopy cover × vegetation cover	**0.4**	**0.02**	**0.79**
	RE year	0.97	0.58	1.64
	RE site	0.78	0.18	3.67

Plot ID, site and year were included as random effects (RE) in all models. An observational level random effect was used in the seed predation and establishment models accounting for overdispersion. Given are standardized effect sizes and 95% confidence intervals (CI) as a measure of support. For REs standard deviations (SD) are shown. Estimates with CIs not passing zero boundaries are considered as significant and highlighted in bold. Sample sizes (n) for each model are given below the response variables.

**Figure 3:**
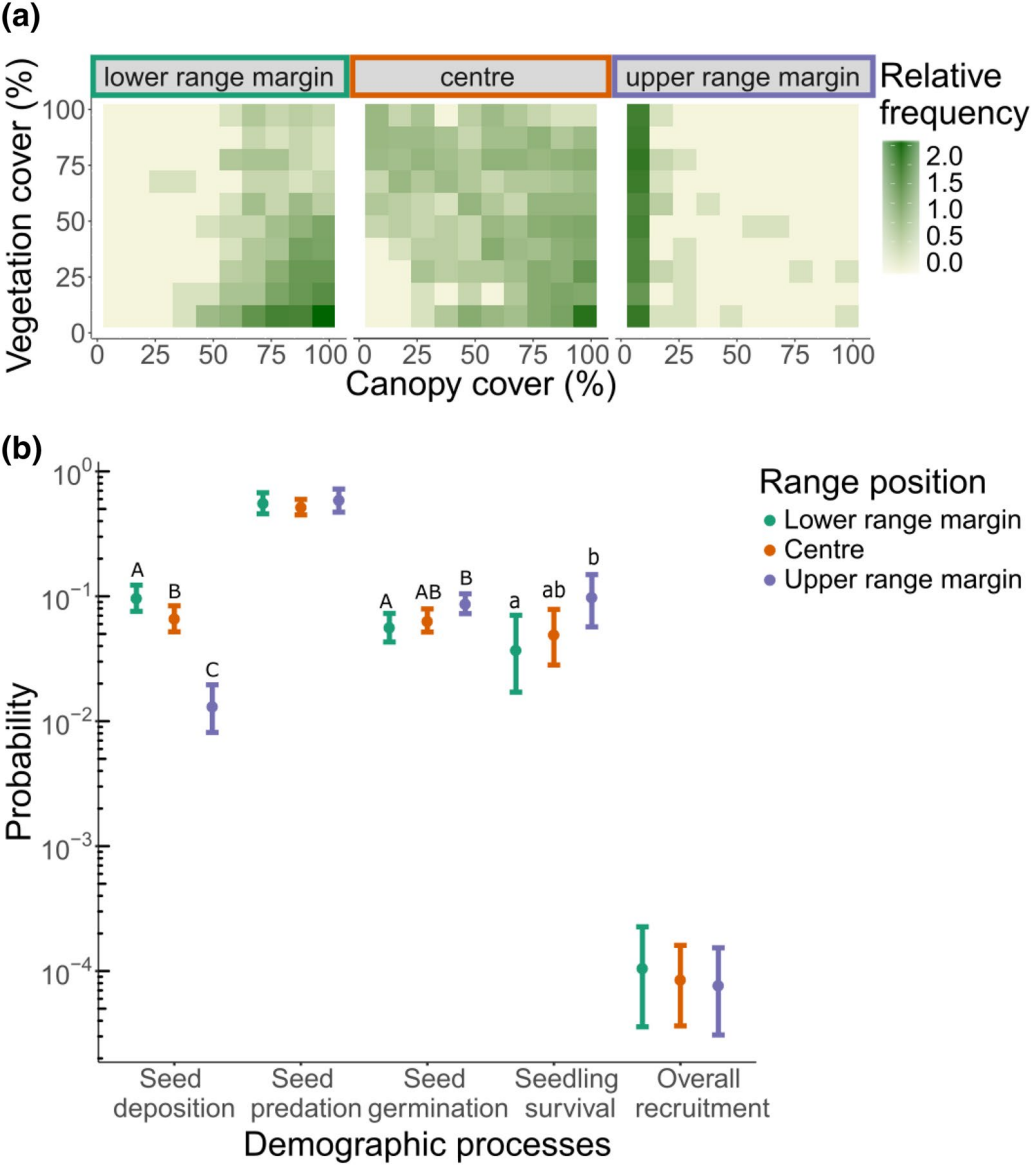
(**a**) Relative frequency (log scale) of environmental conditions in terms of canopy and ground vegetation cover measured at each range position of Swiss stone pine. Frequencies are based on the sum of 1 m^2^ seed deposition subplots with the respective combination of canopy and ground vegetation cover in a 10 × 10 matrix of the values ranging from 0 to 100% in steps of 10%. (**b**) Realized transition probabilities (log scale) of the respective demographic processes and overall recruitment at each range position based on the locally realized environmental context. The realized transition probabilities of seed deposition were significantly different between all range positions (A, B, C). The realized transition probabilities of seed deposition were significantly different between all range positions (*P* < 0.05; indicated by uppercase letters). The realized transition probability of seed germination at the upper range margin was significantly different to the probability at the lower range margin (*P* < 0.05; significant pair-wise differences indicated by uppercase letters), whereas the differences in realized transition probabilities of seedling survival at the lower compared to the upper range margin were marginally significant (*P* < 0.1, marginally significant pair-wise differences indicated by lowercase letters).

We found substantial environmental variation across the species elevational range (Fig. 3a). The majority of microhabitats at the lower range margin (1,850–1,950 m a.s.l.) and at the centre of the elevational distribution (2,000–2,100 m a.s.l.) of Swiss stone pine were characterized by high canopy cover and low ground vegetation cover (Fig. 3a). In contrast, at the upper range margin (2,150–2,250 m a.s.l.) at and above the tree line, microhabitats were predominantly characterized by the lack of canopy cover and high ground vegetation cover (Fig. 3a). Environmental variation across the three range positions resulted in pronounced differences in the realized transition probabilities accounting for the environmental context at each range position (Fig. 3b, Supplementary Table 1). Seed deposition significantly decreased across the range positions by 90% from 10% at the lower range margin to 1% at the upper range margin (*P* < 0.001; Fig. 3b, Supplementary Table 1). Seed predation was overall high and did not differ significantly between range positions (i.e. between 52–60%; Fig. 3b, Supplementary Table 1). Seed germination significantly increased across the range positions from 6% at the lower range margin to 9% at the upper range margin (*P* < 0.05; Fig. 3b, Supplementary Table 1). Seedling survival increased from 4% at the lower range margin to 10% at the upper range margin, but owing to the comparatively small sample size the uncertainty in this trend was relatively large (*P* < 0.1; Fig. 3b, Supplementary Table 1). Overall, the realized recruitment probabilities in the different environments did not differ between range positions and ranged from 0.011% to 0.008% (Fig. 3b, Supplementary Table 1).

We found that the realized transition probabilities of particular demographic processes changed across the species’ elevational range, due to variability in the biotic and abiotic context. As a result, different demographic processes have the potential to limit pine recruitment depending on the range position^12^. Our result suggests that demographic processes related to seed germination and seedling survival were crucial for recruitment at the upper range margin of a plant's distribution, and compensated for low rates of seed deposition at this range boundary. Swiss stone pine depends on a single seed-dispersing bird, the Spotted nutcracker, which extracts pine seeds from closed cones to deposit them in caches beneath the soil surface for later consumption^17^. The bird’s selection of caching sites is driven mainly by two microhabitat characteristics (i.e., canopy and ground vegetation cover)^17,18^. Beyond the tree line, the preferred caching microhabitats of nutcrackers are absent (i.e., high canopy cover, low ground vegetation cover) and the probability of seed deposition is low^18^. Previous work shows that nutcrackers avoid to deposit seeds at high elevations because seeds are more likely to germinate at these sites and since deep snow during winter and a lack of landmarks hinders the recovery of seeds^17^. Thereby, the environmental conditions at the upper elevational range boundary cause a dispersal limitation for Swiss stone pine. Dispersal limitation by animal-mediated demographic processes in peripheral populations have been proposed to be critical bottlenecks when plants are forced to respond to rapid environmental changes^21,22^. Indeed animal-mediated seed dispersal may be highly sensitive to environmental changes, since this plant-animal mutualism depends on a spatial overlap of favorable environmental conditions for both interacting species^23,24^. Moreover, the strength and even the mutualistic nature of the interaction between plants and their animal partners can be context dependent^25–27^. For example, if environmental conditions are altered, this may affect animal seed dispersal through alteration of seed quantity and quality^27^ as well as through context-specific changes in animal behaviour^28^. The context-dependency of animal-mediated demographic processes can have severe impacts on future range dynamics of plants, for instance, because ~ 42% of woody species in temperate coniferous forest and up to 90% in tropical rainforest are adapted to animal seed dispersal^29^. Our findings support the notion that animals play a pivotal role in driving plant range dynamics and determine a plant's capacity to effectively colonize new habitats, especially under the light of rapid climate and land-use change.

At the upper elevational range margin of Swiss stone pine distribution, seed germination and seedling survival increased, likely due to changes in biotic and abiotic conditions driven by low canopy cover at high elevations. Previous work has shown that dark and dry conditions under dense canopy hinder seed germination and seedling survival of Swiss stone pine (Supplementary Table 2) ^17,18^. Furthermore, sites with high pine canopy cover show a high density of specialist fungal pathogens that reduce pine seedling survival^19,20^. Consequently, increased light availability and soil moisture, as well as a reduced frequency of antagonistic fungi at the range margin could result in enhanced seed germination and seedling survival^18, 20^. In contrast, a lack of recruitment at the lower range margin and at the range centre, because of dark and dry conditions under dense canopy and high density of antagonistic fungi, might put Swiss stone pine at a competitive disadvantage against upward migrating plant species. At these range positions, competitive interactions could hamper the persistence of peripheral plant populations at the lower range margin and the persistence at core populations^30^. Importantly, the major demographic bottlenecks of such populations, and the targets for population management^31^, would be distinct from those at the species' upper elevational range.

We conclude that the limiting demographic processes of plant recruitment can vary substantially across the distributional range of a plant. The identification of the limiting plant recruitment processes across a species range is especially relevant for range predictions under climate change^11,32^. Our multi-year experiment shows how the relevance of demographic processes can be assessed for a keystone species of the alpine tree line, and similar experiments could be applied to a range of plant life forms and taxa. In our study case, we reveal that the interplay between biotic interactions and abiotic conditions plays a crucial role in shaping plant recruitment at the range margin and is likely to be pivotal for the future range expansion of the Swiss stone pine.

## Methods

### Study area and design

We conducted this study within the geographical distribution centre of Swiss stone pine in the Central Alps. We chose two elevational gradients close to Davos, Switzerland, encompassing the whole elevational distribution of Swiss stone pine*;* one in the Sertig valley (46°44′0.76″N, 9°51′3.5″E) and one in the Flüela valley (46°48′0.25″N, 09°54′15.38″E). The forest structure at the lowest elevational belts (about 1,850 m a.s.l.) is a mixed coniferous forest, mainly composed of European larch (*Larix decidua*) and Norway spruce (*Picea abies*). The abundance of Swiss stone pine is distributed unimodally from 1,850 m a.s.l. up to 2,150 m a.s.l., where pine trees (> 3 m tall,^33^) form the upper tree line. Young pine trees can be found up to 2,200 m a.s.l., but none are growing at and beyond 2,250 m a.s.l.^18^. In each valley, we established nine elevational belts spaced by 50 m of altitude ranging from 1,850 to 2,250 m a.s.l. reaching across and beyond the elevational distribution of the pine.

### Demographic processes

Seed deposition sites (i.e., seed caches deployed by Spotted nutcrackers) were recorded by randomly selecting a 2 × 10 m plot within each elevation belt. Each 20 m^2^ plot was composed of 20 1 m^2^ subplots, resulting in 20 subplots at each of the nine elevational belts per valley. In the centre of each subplot, we took soil samples (1 dm^3^ per sample) and thoroughly searched for deposited seeds. Intact seeds or seed shells handled by nutcrackers were recorded as cache presence and marked as a seed deposition site. We recorded seed deposition during the main seed-caching season in mid-August until beginning of September over six years (2012–2017), resulting in a total number of 2,156 soil samples. In these soil samples, we found 256 (12%) seed caches deposited by nutcrackers across the elevational gradient in both valleys and all years.

To determine rates of seed predation, seed germination and seedling survival, we conducted a seed transplant experiment across the nine elevational belts. According to a random-stratified sampling design, we selected five microhabitat types at each elevational belt for the experiment (1. soil covered by vegetation, 2. close to adult Swiss stone pine individuals [i.e., up to a distance of 1 m], 3. open soil, 4. rocky habitat, 5. microsite covered by snow [i.e., late snow lie areas]). For elevational belts above the tree line (at 2,250 m a.s.l.), the microhabitat “close to adult Swiss stone pine” was replaced by matgrass (*Nardus stricta* L.) dominated sites, to guarantee an equal sample size in each elevational belt. At the beginning of the growing season (i.e., end of May), we placed Swiss stone pine seeds in a plastic mesh at two to six replicates per microhabitat at each elevational belt, resulting in a total number of 1,980 seed sowing replicates, including 6,858 seeds monitored during the study (for detailed information on the number of replicates deployed per year see Supplementary Table 3). Each mesh (i.e., insect gauze with 1.5 mm holes, open at the top) contained five Swiss stone pine seeds simulating the average number of seeds per cache deposited by nutcrackers^16^. From the total number of 1,980 meshes, 540 meshes were protected by 1.5 mm wire-cages in the field to prevent loss of seeds. Meshes were buried about 4 cm deep in the soil and fixed with metal pins. To break dormancy of the seeds, seasonal variation was simulated in a wet clay-sand mixture by exposure to temperature shifts between 5–25 °C for 22 weeks. At the end of the growing season (i.e., end of September), we evaluated whether seeds had been preyed upon or germinated. Further, we monitored the survival of seedlings until the end of the subsequent growing season in the following year. Out of 6,858 seeds monitored in the six study years 3,023 seeds (44%) were preyed upon and removed by rodents and other animals and 451 (7%) germinated within the first growing season (i.e., the period between May and September). Of 319 seedlings 65 (20%) survived to the end of the following growing season (i.e., the period from September to September of the following year) in two years (i.e., 2014–2015, 2015–2016). We included only these two years of survival as these were the only years where numerous seedlings survived to the next year.

### Environmental variables

Canopy and ground vegetation cover are decisive for seed caching behaviour of Spotted nutcrackers^17, 18^, and they strongly affect seed germination and seedling survival of Swiss stone pine via shading and abiotic soil properties (i.e., soil surface temperature and soil moisture)^18^ as well as the presence of fungal pathogens^19^. Therefore, these two factors do affect both the selection of caching sites and the subsequent demographic processes of plant recruitment of Swiss stone pine. Canopy cover was measured at each seed deposition subplot and seed sowing replicate with a spherical densitometer. On the same sites, ground vegetation cover was assessed by estimating the percentage cover of dominant ground flora species: *Juniperus communis* L., *Loiseleuria procumbens* (L.) Desv., *Vaccinium* spp. L. and *Rhododendron ferrugineum* L. within 1 m^2^^34^. In total, we measured canopy cover and ground vegetation cover at 4,136 1 m^2^ plots, i.e., at 2,156 seed deposition subplots and 1,980 seed sowing replicates.

We characterised the frequency distribution of canopy and ground vegetation cover across three range positions. To do so, we used the data collected at the 2,156 1 m^2^ seed deposition subplots across the 9 elevational belts and categorized these into three range positions: (1) three lowest elevational belts (i.e., lower range margin, 1,850–1,950 m a.s.l.), (2) three central elevational belts (i.e., centre, 2,000–2,100 m a.s.l.) and (3) three high elevational belts (i.e., upper range margin, 2,150 – 2,250 m a.s.l.). The relative frequency of canopy and ground vegetation cover for each range position was determined, taking into account the interaction between the two measures. To this end, each 1 m^2^ subplot was assigned to a cell of a 10 × 10 grid of values for canopy cover and ground vegetation cover ranging from 0 to 100% in steps of 10%. Each combination of canopy cover and ground vegetation cover was counted as the sum of subplots within the respective combination of values.

In addition, we assessed how the microhabitat characteristics (canopy and ground vegetation cover) control microclimatic environmental conditions in terms of soil surface temperature and soil moisture. Soil surface temperature was recorded every four hours over the duration of the study using iButton data loggers (Maxim) at 1,343 seed deposition and seed transplant sites across the elevational gradient and years. We calculated the mean of daily temperatures of the hottest three months of each year (i.e., June, July, August). Soil moisture was recorded on 4,136 seed deposition and seed transplant sites across the elevational gradient and years. Soil moisture was recorded under dry weather conditions in September by averaging five tensiometer (Theta-Kit version 3) measurements at each seed deposition and seed transplant site. Using linear mixed models including canopy cover and ground vegetation cover, we found soil surface temperature during the hottest three months and soil moisture to be negatively associated with high canopy and ground vegetation cover (Supplementary Table 2). Thus, our results suggest that the microhabitat characteristics are the main cause for site by site variation in microclimatic conditions, such as variations in soil surface temperature and soil moisture. Hence, we focussed on environmental variation in canopy cover and ground vegetation cover, as the most appropriate descriptors of the variation in microhabitat conditions across the species range.

### Statistical analyses

We assessed the recruitment probability across and beyond the whole elevational range of Swiss stone pine. To do so, we fitted four models describing the determinants of seed deposition (i.e., the presence or absence of cached seeds in soil samples taken at each subplot), seed predation (i.e., absent seeds and seeds that showed signs of seed predation [e.g., bite marks] at seed translocation sites), seed germination (i.e., seed germination and seedling establishment within the first growing season), and seedling survival (i.e., survival from the end of the first growing season to the end of the second growing season). We used the presence or absence of cached seeds rather than the actual seed number recorded at a caching site to account for potential methodological constraints in assessing the exact number of deposited seeds. We used generalized linear mixed models with a binomial error distribution in the R package ‘lme4′^35^. We included canopy cover, ground vegetation cover and their interaction as continuous explanatory variables into the models. In addition, we included plot ID, site and year as random factors to account for spatial and temporal non-independence. An observation-level random effect was included in the seed predation and seed germination models to account for overdispersion^35,36^. All predictors were mean centred and scaled to unit variance to allow for comparison of effect sizes across predictor variables and models. We used the models to predict the expected probability of seed deposition, predation, germination and first-year seedling survival across the gradients of canopy cover and ground vegetation cover (Fig. 2a–d). Then, we calculated the overall recruitment probability as the product of the expected transition probabilities (*P*) from the four individual models of each demographic process: $P_{{{\text{Recruitment}}}} = P_{{{\text{Deposition}}}} \times \left( {1 - P_{{{\text{Predation}}}} } \right) \times P_{{{\text{Germination}}}} \times P_{{{\text{Survival}}}}$ (Fig. 2e).

We conducted a context-specific analysis to evaluate how the realized transition probabilities of the four demographic processes change at the three different range positions (i.e., lower range margin, centre, upper range margin). To account for the environmental context, we used the realized frequency distribution of canopy and ground vegetation cover at each range position to calculate the weighted mean of the realized transition probability of each demographic process and overall recruitment in the respective environment (Fig. 1). To gain an estimate of uncertainty (confidence intervals) for the realized transition probabilities, we performed a bootstrap analysis with 1,000 replicates. Bootstrap replicates were used to test for significant pairwise differences in realized transition probabilities between range positions (based on two-tailed *P*-values). Bonferroni correction was used to adjust *P*-values for multiple comparisons.

## Supplementary information

Supplementary information.

## Data Availability

Data from this paper are deposited in the Dryad Digital Repository 10.5061/dryad.xsj3tx9c2 (Merges, Albrecht, Böhning-Gaese, Schleuning & Neuschulz, 2020).
