# Supplementary material for: Environmental context determines the limiting demographic processes for plant recruitment across a species’ elevational range
Source: Sci Rep. 2020 Jul 2;10:10855. doi: 10.1038/s41598-020-67602-5 (PMC7331732; doi:10.1038/s41598-020-67602-5)
Supplement: Supplementary file 1 — Supplementary information. [file 41598_2020_67602_MOESM1_ESM.pdf]

Supplementary Information

Environmental context determines the limiting demographic processes for plant recruitment across a species' elevational range

Dominik Merges[1,2], Jörg Albrecht[1], Katrin Böhning-Gaese[1,2], Matthias Schleuning[1], Eike Lena Neuschulz[1]

[1] Senckenberg Biodiversity and Climate Research Centre, Frankfurt am Main, DE

[2] Department of Biological Sciences, Goethe Universität Frankfurt, Frankfurt am Main, DE

Supplementary Table 1: Summary of the realized transition probabilities of each demographic process of plant recruitment for three range positions (lower and upper range margin and centre of the elevational range), accounting for the environmental variability in terms of canopy and ground vegetation cover across the species' range. Shown are the realized transition probabilities with 95% confidence intervals (CI) and Bonferroni corrected two-tailed *P*-values as a measure of statistical significance. Significant values are highlighted in bold.

**Process**	**Range position**	**Realized transition probability**	**Lower CI**	**Upper CI**	***P* Difference to lower**	***P* Difference to centre**
Seed deposition	lower	0.0989	0.0758	0.123	/	/
	centre	0.0677	0.052	0.0839	< **0.01**	/
	upper	0.0134	0.00813	0.0196	< **0.01**	< **0.01**
Seed predation	lower	0.567	0.458	0.674	/	/
	centre	0.525	0.448	0.597	0.798	/
	upper	0.6	0.471	0.722	1	1
Seed germination	lower	0.0576	0.0431	0.073	/	/
	centre	0.0647	0.0517	0.0792	0.282	/
	upper	0.089	0.0726	0.105	**0.030**	0.054
Seedling survival	lower	0.038	0.0171	0.0705	/	/
	centre	0.0504	0.0282	0.0786	0.330	/
	upper	0.1	0.057	0.149	0.066	0.090
Overall recruitment	lower	0.000108	0.0000358	0.000226	/	/
	centre	0.0000872	0.0000364	0.000161	1	/
	upper	0.0000785	0.0000308	0.000154	1	1

Supplementary Table 2: Summary of linear mixed models testing the effects of canopy cover, vegetation cover and their interaction on factors potentially important for recruitment processes (i.e. mean soil temperature, mean soil moisture). The sample size (n) results from sites where respective variables were measured on a microhabitat level. Year and site were included as random factors in all models. Shown are effect estimates with 95% confidence intervals (CI) as a measure of support. For REs standard deviations are shown. Estimates with CIs not passing zero boundaries are considered as significant and highlighted in bold.

Response	Variable	Estimate	2.5% CI	97.5% CI
**Mean soil temperature** n_{obs} = 1343, n_{year} = 6, n_{site} = 18	Canopy cover	**-0.83**	**-0.95**	**-0.72**
	Vegetation cover	**-0.09**	**-0.17**	**0**
	Canopy cover* vegetation cover	**0.1**	**0.02**	**0.17**
	RE year	0.48	0.34	0.72
	RE site	1.09	0.62	2.01
**Mean soil moisture** n_{obs} = 4136, n_{year} = 6, n_{site} = 18	Canopy cover	**-11.31**	**-12.25**	**-10.36**
	Vegetation cover	**-1.17**	**-1.81**	**-0.54**
	Canopy cover* vegetation cover	**3.19**	**2.59**	**3.8**
	RE year	7.93	5.71	11.43
	RE site	5.58	3.21	10.71

Supplementary Table 3: Summary of seed translocation experiment conducted over a 6 year period. "Elevation" refers to elevation in m a.s.l.. "Treatment" refers to an open treatment (mesh) without protection from predation or to an exclosure treatment where seeds were protected from predation by a wire-cage. "Overall seeds" as product of "Seeds per mesh", "Replicates", "Micro-habitats", "Elevational belts" and "Valleys".

**Year**	**Elevation**	**Treatment**	**Seeds per mesh**	**Replicates**	**Micro-habitats**	**Elevational belts**	**Valleys**	**Overall replicates**
2012	1850-2250	open	5	2	5	9	2	180
2013	1850-2250	open	5	4	5	9	2	360
2014	1850-2250	open	5	2	5	9	2	180
	1850-2250	exclosure	5	2	5	9	2	180
2015	2100-2250	open	5	6	5	4	2	240
	1850-2250	exclosure	5	4	5	9	2	360
2016	2100-2250	open	5	6	5	4	2	240
2017	2100-2250	open	5	6	5	4	2	240
							Total =	1980
